# Maintaining Health Care in Occupied Ukraine: Criminal Collaboration or Conscientious Professionalism?

**DOI:** 10.1002/hast.70005

**Published:** 2025-09-11

**Authors:** Michael L. Gross

**Keywords:** wartime collaboration, Russian‐Ukrainian war, military medical ethics, international humanitarian law, military occupation, belligerent occupation, public health

## Abstract

*Following Russia's occupation of Eastern Ukraine, local health care professionals, particularly hospital administrators and public health officials, have faced criminal charges of medical collaboration for taking senior managerial positions in the occupation regime and allocating resources to the Russian army. Although Ukraine is entitled to prosecute collaborators who threaten its national security, the grounds for criminalizing medical administration during military occupation are much weaker and essentially indefensible. During occupation, international humanitarian law requires Russia to maintain adequate health care services with the assistance of local officials, protects these officials from prosecution, and ensures their independence as they weigh their professional duties to maintain essential health care for the civilian population. An analysis of representative legal cases charging health care professionals with treason and collaboration demonstrates the shortfalls of Ukrainian policy that (a) does not clearly differentiate between charges of collaboration, aiding and abetting the enemy, and treason, (b) rejects public health officials’ duty to cooperate with an occupation regime, and (c) ignores health care administrators’ right to condition their decision to cooperate on its attendant costs and benefits. Recognizing a policy of* humanitarian cooperation *rectifies these deficiencies by highlighting the independence of health care officials, their protection from prosecution by Ukrainian and Russian authorities, and each party's duty to maintain adequate medical care for the local population during military occupation*.

## Neuroscience & Society

Following the Russian invasion and occupation of eastern Ukraine, Russia faced the urgent need to maintain local health care services. By international humanitarian law, the Geneva Conventions require occupying powers to provide adequate medical services to civilian populations. At the same time, ensuring access to health care amid the turmoil of war is strategically beneficial, as it helps to pacify the local population and solidify support for the occupying regime. To these ends, Russian officials seized health care facilities and recruited and coerced local providers and health officials to supply uninterrupted medical care.

Aware that an occupying power uses public services to entrench its rule over conquered territory, Ukraine immediately enacted legislation to severely punish citizens who collaborated with an “aggressor state” or its occupation administration. Ukraine's interpretation of criminal collaboration, or “collaborationism,” is sweeping. It implicates citizens who endanger Ukrainian security by serving in Russian paramilitary forces, transferring Ukrainian resources to Russian‐occupied territory, or otherwise colluding with Russian authorities. The legislation concerning collaborationism also targets public officials who voluntarily assume administrative and managerial positions within local government departments now operated by the occupying power.^1^ Unlike collaboration motivated by pecuniary or ideological reward, administrative collaboration ensnares judges, municipal officers, and health care administrators who continue to perform their official duties during occupation.^2^ Referring to these officials, the Ukrainian government took a severe line. “We are understanding of people who must continue to work: doctors, utility workers, etc.,” declared Mykhailo Podolyak, advisor to the head of the Presidential Office, “but not of [administrative] collaborators in power. There will be no mercy for them.”^3^ Punishments included substantial fines, loss of all employment opportunities (“lustration”), forfeiture of property, and imprisonment.

Penned in by Russian authorities and Ukrainian law, health care professionals—particularly those managing medical facilities in the occupied territories—face a profound dilemma: defy Ukrainian law and continue to supply medical services to the population of occupied Ukraine or abide by the law, refuse to support the Russian regime, and abandon their professional duties. In practice, health care professionals have done both. Some have resigned, while others have stayed on, often replacing those who have fled the occupation. In response, Ukrainian authorities investigated and prosecuted health care professionals, including clinical medical workers, public health officials, and hospital administrators, on charges of treason and collaboration. Through February 2025, twenty clinical and nonclinical professionals stood trial. The number of those investigated or arrested and not yet tried is unknown. Occasionally, these investigations appear in the press.^4^ District Court decisions are accessible through the Unified State Register of Court Decisions, where many defendants were tried in absentia.^5^

Charges of medical collaboration raise several interrelated dilemmas for health care professionals, stemming from three sets of competing legal, bioethical, and civic norms. First, international humanitarian law (IHL) requires occupying powers to recruit local medical workers to ensure the provision of health care services. However, IHL also permits public health officials to refuse, on the grounds of conscience, to cooperate with an occupying regime. They may, therefore, conscientiously choose to serve or oppose the occupying regime, thereby maintaining or impairing access to health care. Second, professional duties of care, including beneficence and impartiality, demand strict attention to patient welfare, reinforcing public health officials’ duty to remain on the job. Finally, civic duties require allegiance solely to one's state and support the state's concomitant right to criminalize wartime collaboration and prosecute health officials. In contrast to professional duties of care, civic duties buttress medical administrators’ right to refuse their cooperation.

The following sections clarify these legal, bioethical, and civic norms and the dilemmas they generate when Ukraine prosecutes health care professionals for activities classified by law as treason, administrative collaboration, and providing clinical support to the occupation regime. In most cases, I will argue, Russian policy and Ukrainian law impose indefensible hardships on medical professionals. Medical cooperation with an occupying power represents a humanitarian duty and not collaboration or treason. Therefore, health care professionals’ efforts to fulfill their duties under the strains of occupation should be met with forbearance rather than criminal prosecution.

## Legal Duties to Maintain Health Care Service during Military Occupation

Two broad legal rights and correlative duties underlie health care maintenance in an occupied territory. To safeguard the rights of the civilian population, IHL first charges the occupying power with “the duty of ensuring and maintaining, with the cooperation of national and local authorities, the medical and hospital establishments and services, public health and hygiene in the occupied territory.”^6^ To this end, an occupying power may lawfully compel essential medical workers to provide services necessary for the health of the local population.^7^ However, occupation authorities cannot forcibly retain essential workers and cannot impose sanctions or other coercive measures when public officials, including public health officials,^8^ “abstain from fulfilling their functions for reasons of conscience.”^9^ Second, IHL recognizes the right of public officials to professional independence and protects them from duress by the occupying regime and from prosecution by their home government. Public officials must be free to continue to perform their public duties under occupation and “not run the risk of being called to account for disloyalty when the national authorities resume their rights after the occupation ceases.”^10^ In other words, Ukrainian health officials in Russian‐occupied territory should be able to choose to fulfill or reject their public responsibilities without fear of retribution from Ukraine or Russia.

Although the laws protecting the professional independence of public officials are unequivocal, forces on the ground are not charitable. The Russians maintain public services to pacify the local population and cement their regime, and to achieve that end, they have intimidated and coerced health care workers and administrators.^11^ Propelled by Ukraine's history of collaboration, Ukrainian legislation criminalizes health care and other public administrators working in the occupied territories, transforming their right to refuse cooperation into a firm, civic obligation that they must fulfill on pain of punishment. Neither Russia nor Ukraine is eager to acknowledge the right of public officials to fulfill or renounce their professional duties as their conscience dictates.

A conscientious decision to support or defy the regime may first appeal to respect for humanitarian law. Public officials are rightly censured for collaborating with a manifestly criminal regime that commits war crimes and crimes against humanity, including wholesale murder, enslavement, genocide, ethnic cleansing, and torture.^12^ Russia stands accused of numerous war crimes since 2022.^13^ In the health care sphere alone, Russian authorities are charged with misappropriating health care facilities for military purposes, arbitrarily obstructing civilian access to medical services, and abusing health care workers.^14^ However, opposing the regime to undermine its control of Ukrainian territory may harm patients’ welfare as the health care community withdraws its services. Under these circumstances, exercising one's conscience is as much about respect for humanitarian law as about weighing one's conflicting professional and civic duties.

## Professional Duties and the Obligation to Provide Care under Occupation

What professional medical obligations drive health care workers to serve the community in an environment where health care access is poor and medical personnel face constant threats? The Russian occupation of Eastern Ukraine has wreaked havoc on the local health care system, significantly impaired access to medical care, and endangered staff.^15^ The advocacy group Physicians for Human Rights describes how numerous health care facilities were damaged or destroyed, while others were expropriated by the military or stripped of essential equipment and medicines to serve the Russian army at the expense of local civilians. Following the Russian takeover, many Ukrainian citizens were subject to “forced passportization” and compelled to take Russian citizenship to qualify for health care. Wartime drug shortages have pushed costs beyond the reach of many who are already suffering from war. Insecurity, poor transportation infrastructures, and a lack of proper documentation have further hindered access to care. Throughout all this, Ukrainian doctors have been harassed, detained, and sometimes tortured and killed.^16^

Health care professionals’ duty to risk their lives to care for the sick and injured is an enduring moral challenge during war and peace.^17^ One gynecologist practicing in occupied Ukraine asked pointedly, “Who is the bigger traitor—the one who left, or the one who stayed to save our citizens? The number of births in our city has not decreased. At the beginning of the war, I, like other medics, had no moral right to abandon people during air raids.”^18^ Typically, dilemmas of providing care in a high‐risk environment stem from the dangers of infectious disease, such as AIDS, Ebola, and Covid‐19. Once considered a near‐absolute clinical norm, the obligation of health care professionals and medical administrators to provide care at substantial personal risk has eroded.^19^ Restoring a stronger duty to treat under high‐risk conditions depends on acknowledging medicine's occupational hazards, mitigating risk through vaccines and protective measures, offering financial incentives to volunteers, and maintaining a robust social contract that holds public health officials accountable for serving society and holds clinicians responsible for treating those in medical need.^20^

During armed conflict, the threat to medical personnel comes not only from disease but also from acts of war, from which personnel can suffer both lawful collateral harm and unlawful ill‐treatment. In addition to the undetermined number of those arrested and detained, 208 health workers and patients were killed and 720 injured in Russian attacks from February 2022 to March 2025.^21^ While the foregoing suggestions to mitigate the risks of infectious disease are promising, most do little to ameliorate war‐related risks confronting health care professionals. Protective measures (such as shielding hospital wards from bombs) and financial incentives (similar to combat pay) are often unavailable in war‐torn nations. Ukraine opened its first of several planned underground hospitals only in September 2024.^22^ While clinical and administrative health workers must recognize the occupational hazards of their profession, no profession but soldiering prepares its members for the perilous conditions of armed conflict.

Armed conflict further tempers the social contract undergirding health care provision. First, war may circumscribe the moral duties of care more than some pandemics do. When risks are relatively moderate, write Abigail Zuger and Steven H. Miles concerning the hazards of treating HIV, they might be met with “courage and intellectual integrity.” But when risks are significantly greater, as happens during devastating epidemics, they “evoke virtues on the order of heroism, self‐sacrifice, and daring.”^23^ The dangers of armed conflict in Ukraine are similarly extreme. As a result, treating patients under fire or occupation is often beyond the call of duty and is not necessarily morally incumbent upon health care professionals. Second, health care professionals, no less than other citizens, are parties to additional social contracts that create civic obligations to serve and protect their body politic, which clash fiercely with the duties of beneficence during occupation.

## Civic Duties during Occupation

Under the challenging circumstances of occupation, nations like Ukraine enact laws criminalizing treason and collaboration to deter their citizens from aiding their enemy. These concerns are weighty and speak directly to a citizen's paramount duty of allegiance to a well‐ordered, rights‐respecting state, particularly during war. Sustaining health care services under occupation, however, is not politically benign. No less than any other public welfare institution, institutionalized medical care permits the occupying power to consolidate its regime; impose judicial, social, and cultural norms; enlist support from local civilians through goods and services; collect taxes for further operations; and usurp territory. Ukrainian health care professionals further enhance Russian military capabilities by providing medical support and resources for their military personnel and health care facilities. As a result, charges of collaboration are not groundless. Nevertheless, Ukrainian demands that health care workers do their utmost to resist the occupying regime depreciate their principal obligation to deliver professional medical services.

While Ukrainian officials have no interest in fortifying the occupation regime and may interpret any form of cooperation as a security threat, they cannot overlook how IHL requires some degree of regime entrenchment so that the occupation regime can provide essential services and ensure adequate medical care.^24^ “Adequate” health care, though not defined in IHL, coincides with “essential” health care, which is measured on a moving scale that depends on local conditions. Still, adequate health care entails providing a minimal set of health care services and essential drugs while furnishing the “underlying determinants of health, such as access to potable water, adequate sanitation, nutrition, healthy occupational and environmental conditions, and access to health‐related education and information.”^25^ The obligation to maintain these essential medical services falls on the Russian occupation regime, local public officials, and Ukrainian authorities. The grounds for prohibiting compliance with Russian authorities are therefore weak, insofar as the regime ensures vital services and abides by IHL protecting civilians and health care professionals from ill‐treatment. Against this, Ukrainian authorities may prosecute high treason and related security offenses to sustain their war effort.

Unequivocally equating collaboration with providing medical care to the Russian military is similarly problematic. International law and ethical medical practices do not offer definitive guidance about the duty to treat enemy military personnel during military occupation. On the one hand, the same international norms that allow an occupation regime to compel workers to deliver essential services also exempt these workers from providing services that benefit the occupying military forces.^26^ Civic duties, including loyalty, fidelity, and allegiance to the home state, bolster this exemption. On the other hand, IHL and civilian medical ethics carve out a special obligation that requires medical personnel to treat the wounded impartially, solely based on medical need and without regard to national identity.^27^ These norms conflict. They charge clinicians and administrators with the duty of impartial care yet grant any occupied civilian the right to refuse aid to Russian military personnel.

Whether this right to refuse aid extends to denying an enemy medical care depends on the level of wartime risk exposure. These risks may be exceptionally high. Compared to health care workers laboring under the risk of infectious disease during a pandemic, health care workers providing medical support during occupation confront an entirely different, nonmedical set of dangers. First, they face the threat of death and injury in combat, coupled with abduction, detention, and ill‐treatment by the occupying regime.^28^ Second, they must consider the likelihood that their services will aid and abet the occupying power, fortify the regime, and undermine Ukraine's war effort, particularly if they are tasked with providing medical resources for the Russian military. Under these circumstances, medical workers, public health officials, and hospital administrators must weigh personal costs (detention, torture, injury, and death) and collective costs (eroding Ukrainian sovereignty and enhancing Russian security in the occupied territories) against the benefits of securing their public's health. Each cost and benefit is an integral part of conscientious decision‐making. Nevertheless, and not withstanding Ukrainian law, the obligation to vigorously oppose a military occupation at significant risk should be less compelling than fulfilling health care workers’ professional and public duties. For the same reasons that diminish the obligation to treat patients under conditions of extreme risk, the duty to extirpate an occupying force falls on the home government and its military, not the occupied civilian population. Civilian resistance is heroic and, by definition, supererogatory, an act worthy of acclaim but not punishable by law in the breach.

In summary, three principles evaluate alleged criminal collaboration among health care professionals. First, IHL stipulates the rights and obligations of the occupying power, the home government, civilians, and public officials. On the one hand, IHL requires the occupying regime to ensure health care services and permits it to forcibly employ health care workers. On the other hand, IHL prevents an occupying regime and the home government from punishing public officials, including health care administrators, who follow their conscience as they decide whether to cooperate with the occupation officials. Second, subject to reasonable risk, medical ethics imposes the duty to provide impartial, beneficent care for local civilians and enemy soldiers alike. Third, civic norms encourage dissent and disobedience but excuse cooperation when the personal and collective dangers of failing to comply with the regime are considerable. Whether clinicians and health officials affirm their pivotal role to maintain public health or appeal to the greater good of undermining an occupying regime, IHL allows them to justifiably equivocate as they decide whether to cooperate with the regime.

Despite the protection of international law, Ukraine has prosecuted health care professionals for high treason, administrative collaboration, and providing medical support in Russian‐occupied territory. Although each offense turns on betraying the state and collaborating with the enemy, they differ significantly in the actions they criminalize, the punishments they impose, and the dilemmas they raise. Considering each offense in order of severity illuminates how humanitarian, professional, and civic norms should inform moral decision‐making under occupation.

## Treasonous Collaboration during the Russian Occupation

Treason comprises willful actions to undermine Ukraine's sovereignty, security, or territorial integrity and may afford the home country a compelling reason for prosecuting its health care professionals in occupied territories.^29^ Nevertheless, conceptual difficulties arise that sometimes overemphasize fidelity to the state at the expense of respecting humanitarian norms and professional obligations. Consider two representative cases.

- **Treason 1:** Valentina Chekhova, an ophthalmologist at a hospital in Mariupol, Ukraine, voluntarily and knowingly passed on information about seriously wounded Ukrainian soldiers hospitalized in her facility to the Russian military during an inspection of the hospital between March 12 and 15, 2022. The Russians took the Ukrainian soldiers into captivity, where they suffered “physical and moral abuse.” The Ukrainian court convicted Chekhova of treason and sentenced her to life imprisonment in absentia for assisting a foreign state in conducting subversive activities against Ukraine.^30^
- **Treason 2:** Oleksandr Pasternak, head of the geriatric department of City Hospital No. 9 in Mariupol, was charged with treason for participating “in staged videos of Russian propagandists that justified the aggression of the Russian Federation and discredited the Ukrainian Armed Forces.”^31^

Chekhova committed multiple betrayals. Divulging a patient's identity compromises confidentiality. Turning in soldiers who might well suffer abuse violates their human rights and the doctor's ethical duty of “do no harm.” Denouncing compatriots disregards one's civic obligations to shield one's soldiers who might sufficiently recover to rejoin their forces and continue fighting the enemy. Each betrayal bears a different weight. IHL and fundamental human rights generate the strongest duties. Nevertheless, captors have a legitimate military interest in identifying wounded enemy soldiers. IHL permits them to invoke military necessity and compel medical personnel to supply information about the sick and wounded under their care unless “such information would, in [medical personnel's] opinion, prove harmful to the patients concerned or to their families.”^32^

Several key ideas imbue these norms. First, prisoners of war (POWs) retain the inviolable right to humane treatment. Second, military necessity permits warring parties the right to apprehend and incarcerate enemy soldiers. But limited to the legal means necessary to pursue a legitimate military purpose, military necessity cannot override POWs’ fundamental human rights that guarantee respect for dignity and freedom from torture and ill‐treatment.^33^ Only when assured that captors comply with IHL may medical workers be compelled to denounce their soldier‐patients by providing “information about [their patients’] activities, connections, position, or existence.”^34^ However, once afforded sufficient evidence that their patients will suffer harm and mistreatment, doctors have an overriding obligation to shield the patients’ identities.

Nonetheless, the threat of harm is not dispositive. The Geneva Conventions acknowledge that medical workers may denounce the wounded “even when they know that this will be prejudicial to the wounded person or his family if such denunciation is necessary for saving lives.”^35^ Whose lives counterbalance their patients’ welfare is not specified. Presumably, it might include the lives of medical workers, colleagues, their families, or compatriot soldiers. Ultimately, medical personnel are charged with a cost‐benefit calculation that they must later defend against charges of treason. Under these conditions, patient confidentiality is a secondary issue that military necessity or grave threats to others may override.^36^

Chekhova's case does not delve into these questions. Tried in absentia, she offered no defense of her actions, and it is unclear what defense the court would accept. Ruling out any mitigating factors such as coercion, the court found that Chekhova disregarded her obligation to protect the soldier‐patients and failed to conceal their identities, as others in the hospital tried to do. While denunciation alone serves as evidence of subversive activities, might the court have considered an IHL‐sanctioned defense, namely, that denouncing her patient‐soldiers would save lives? Chekhova may have thought it would. She “did not hide her actions from the medical staff,” wrote the court, and she explained “her cooperation with the occupiers with Russian propaganda rhetoric: ‘How much longer will they [Ukrainian soldiers] hide behind our backs? They bombed Donbas for eight years.’”^37^

Chekhova was a Russian sympathizer, and the fighting in Eastern Ukraine after 2014 (including in Donbas) saw the destruction of civilian infrastructures attributable to both sides.^38^ A reasonable fear that hidden and later repatriated Ukrainian soldiers will return to battle and threaten civilians’ lives might motivate a physician who sympathizes with Russia to denounce her patients. Accepting this defense, however, depends upon credible assurances that the soldiers would not suffer mistreatment. Testimony that the denounced soldiers subsequently suffered physical abuse and humiliation strongly suggests this was not the case.

A comprehensive evaluation of treason requires more information—namely, the risk of patient abuse by Russian authorities and the personal risk of noncompliance by hospital staff. Given the probability of patient abuse, Chekhova's professional obligations require her to assume reasonable risk to protect her patients. However, this obligation to protect patients is no more absolute than the duty to take substantial risks to treat patients suffering from infectious diseases. Defying local military authorities by shielding wounded compatriots may require extraordinary heroism, and it is not obligatory if it involves a significant cost to the physician. Under high‐cost circumstances, failing to shield is not a treasonable offense, and the courts are remiss if they do not address this possibility. In contrast, guarantees of humane prisoner treatment (probably absent in this case) may justifiably trigger a clinician's professional obligations to disclose their patient's identity, maintain the integrity of medical services in an occupied territory, and resist the call to actively oppose the occupation regime.

Free of these complications, treason 2 is significantly different from the first case. In treason 2, the doctor was charged with participating in staged Russian videos that defended the Russian invasion and discredited the Ukrainian Armed Forces.^39^ These charges involve no patients, no violations of international humanitarian law, and no direct harm to others. The fact that the perpetrators were physicians has no direct bearing on this case. However, physicians, like educators, enjoy a unique level of trust.^40^ Their propaganda efforts on behalf of the occupation authorities may be more influential and detrimental to the home government than others, which is perhaps why they are doggedly prosecuted by the Ukrainian government. Prosecutors were equally wary of administrative cooperation but did not always draw a clear line between collaboration and treason.

## Administrative Collaboration during the Russian Occupation

Administrative collaboration is the most frequent charge against physicians and senior medical professionals who assume duties as hospital administrators and public health officials in the occupied territories. In these cases, the defendants are not solitary health care workers treating individual patients but vital institutional cogs whose participation rises to the level of voluntarily assuming a managerial role in the occupying regime. Nominally a lesser charge than treason, administrative collaboration is sometimes accompanied by more severe charges, including aiding and abetting the enemy and treason.

- **Administrative collaboration 1:** In Melitopol, Ukraine, an unnamed doctor voluntarily put forward his candidacy for chief doctor and director of the Zaporizhzhia Regional Hospital, ousted the current director, established a hospital for Russian soldiers, and provided them with medicine.^41^
- **Administrative collaboration 2:** In Berdyansk, Ukraine, an unnamed health official was appointed head of the health care department and entrusted with structuring and staffing health care institutions, providing medical and sanitary assistance for the city, treating the local population and Russian Federation military personnel, and cooperating with the media.^42^
- **Administrative collaboration 3:** In Melitopol, an unnamed defendant was appointed chief physician of the occupation‐run Melitopol Primary Health Care Center, met with Russian officials to discuss opening health care facilities, requested material assistance, reorganized primary health care units, recruited personnel, updated unit heads about new work conditions, and implemented Russian regulations. Also charged with treason and “defecting to the enemy,” the defendant “carried out subversive activities against Ukraine by imposing an administrative model of management and standardizing the activities of bodies and institutions of the health care system … to achieve socio‐cultural, humanitarian, informational, economic, medical and other isolation of the [occupied] population.”^43^

Punishments in these cases varied. Some defendants (in cases 1 and 2) were charged under part 5 of article 111‐1 of the Criminal Code of Ukraine and sentenced, in absentia, to five to ten years in prison, ten to fifteen years of lustration, and property forfeiture.^44^ However, a substantial number of the convictions since July 2024 charged medical administrators under the more serious statutes of aiding and abetting the enemy (article 111.2) and treason (article 111).^45^ Here, the punishments included longer prison terms of ten to fifteen years as well as property forfeiture and lustration.

These and other cases share several characteristics. When examining how public officials fulfilled their duties, the courts first sought to establish voluntary cooperation. While extreme physical and psychological pressure (such as immediate threat of detention, torture, or execution) belies voluntariness and collaboration, the courts relied primarily on assurances from witnesses that defendants appeared uncoerced. The bar to establish voluntary cooperation was not high. Because prosecutors tried most cases in absentia, the accused rarely had the opportunity to testify about their motivations for cooperating. Moreover, Ukrainian legal experts and judges disagree about whether and how the exigencies of everyday life under occupation and “future risks” to oneself and one's family excuse collaboration.^46^ As a result, the defendants had little basis for claiming they were coerced.

Second, the court transcripts often introduce elements of moral opprobrium. In these and similar cases, hospital administrators either continued in their existing positions or took new and sometimes more lucrative positions with the occupation authorities when others resigned. As a result, defendants were accused of opportunism, financial greed, obeisance, and rank self‐interest as they maneuvered “to advance up the career ladder.”^47^ While some commentators argued that self‐aggrandizement was sufficient to establish criminal liability,^48^ obsequious, opportunistic behavior, however repugnant, should not warrant criminal prosecution unless it is accompanied by harm to others or malfeasance. Self‐seeking behavior is, at best, only a clear signal of voluntary cooperation, a necessary but insufficient condition to prove collaboration under the law. “A person performing collaborative activities,” writes one Ukrainian District Court judge, “must not only [a] knowingly and voluntarily cooperate with the enemy but must also [b] act in the enemy's interests and [c] to the detriment of his state.”^49^

To argue that defendants met these additional conditions, prosecutors carefully described the defendants’ health care institutional responsibilities, including medical care and services, public health administration, staffing, supply, and supervision. Prosecutors, though quick to cite these activities as evidence of voluntary cooperation, took little note that these same activities demonstrated health officials’ efforts on behalf of the public's welfare, precisely the sort of endeavor international law requires. Noting exemplary public service in one case, the courts credited one experienced health administrator for “signing and approving decrees to ensure the effective functioning of the health care sector in the so‐called ‘liberated’ territories’” and building a health care system that, without her “considerable experience and knowledge,” would have been completed much more slowly. However, these accomplishments did little to mitigate her punishment. Fulfilling her duties was not evidence of professional fidelity but proof of collaboration and support for the occupation regime and brought a stiff sentence of imprisonment, lustration, and loss of property.^50^ In the same vein, a pharmacist who organized the distribution of surplus medicine to civilians was arrested and held for trial on charges of collaboration despite her efforts to aid the local population.^51^

In the third case above, the chief doctor met with Russian officials to request assistance, reorganized the medical units to conform to Russian regulations, and assembled unit heads to introduce their new work conditions. Although these activities were consistent with administrators’ legal and professional obligations, the doctor was charged with subversion, defection, and high treason. While the circumstances of this case are similar to those in the first two cases, the harsher charges and sentence represent a marked escalation in the prosecution of medical collaborators since July 2024.

Despite these severe charges, none of these court cases suggest that administrators violated international or human rights law. On the contrary, health care managers sustained Russia's humanitarian obligation to furnish adequate health care services for the civilian population while fulfilling their professional duties as public officials to ensure the public welfare. While IHL charges occupation powers with maintaining health care services, the Geneva Convention commentators entrust the implementation of these services to local public health officials: “There can be no question of making the Occupying Power alone responsible for the whole burden of organizing hospitals and health services and taking measures to control epidemics. The task is above all one for the competent services of the occupied country itself.”^52^ None of those service providers’ efforts, therefore, constitutes criminal collaboration; instead, the efforts align squarely with humanitarian‐sanctioned cooperation.

Ukrainian charges that the hospital director in the third case “imposed an administrative model of management and standardized the activities of bodies and institutions within the health care system” are not wrong; they precisely encapsulate health care administrators’ legal and ethical obligations during occupation. The conclusion that these measures “achieved the socio‐cultural, economic and medical isolation of the [occupied] population” is not a misplaced concern. Rather, it describes an inevitable byproduct of maintaining medical care under occupation—not a punishable offense. Ukrainian authorities are right to fear the loss of territory as Russia consolidates its hold. However, refusing medical cooperation will not win it back; this only immiserates the populace. Only in the most superficial sense, and by ignoring professional and legal obligations under occupation, can the courts justifiably conclude that health care administrators in cases such as these act “in the enemy's interests and to the detriment of their state.”

Finally, consider criminal charges of collaboration for providing medical resources that sustain the occupier's military capabilities. In response to charges that medical care for Russian soldiers enhances enemy interests to the detriment of Ukraine, some Ukrainian legal experts invoke health officials’ duty to provide impartial care—a duty they shoulder no less than clinicians.^53^ However, because health care administration differs from direct patient care, the duty of impartiality alone is insufficient to exculpate public health officials from charges of collaboration. Administrators do not care for patients; they formulate and implement policies that allocate scarce, vital resources. If those resources reach the Russian military, charges of collaboration or aiding and abetting can ensue. Whether these charges are justified depends on the underlying criteria of allocation. In civilian settings, health care administrators may face criticism for discriminatory or unfair policies. Suppose an allocation scheme favors a particular ethnic community or the wealthy. In that case, administrators have moral grounds to recalibrate allocations by denying care to members of the previously favored community, even if some members require urgent treatment. Fairness and justice, therefore, temper the demands of impartial medical need.

In war, military necessity may temper and override medical needs so that an army medical unit may use resources to return soldiers to duty while leaving critically ill soldiers or civilians to suffer.^54^ Russia may, therefore, shortchange its citizens by transferring resources from the public sector to the military. But Ukrainian civilians in occupied territory have a special right to adequate health care. Understood as essential medical services, adequate care is the benchmark for evaluating the actions of public health officials vis‐à‐vis the occupying regime. Health care administrators criminally collaborate when they support occupation policies, such as a policy of large‐scale transfer of resources to the enemy, that diminish care for the local population to below the threshold of adequacy.

Supporting Russian military medicine, therefore, is not collaborative unless it significantly undermines health care for local Ukrainian civilians. Once health officials have guaranteed essential care for the populace, they should be able to exercise their professional judgment about whether to care for Russian soldiers or allocate resources to their military without facing charges of collaboration or treason for these decisions. Both Ukrainian and Russian authorities exceed their moral reach. The Ukrainian government condemns officials who administer health care services that benefit the Russian military. The Russians persecute officials who conscientiously refuse to aid the occupation when it fails to maintain adequate civilian health care or violates international law.

## Medical Collaboration by Service Providers

In addition to unjustly charging health officials with treasonous and administrative collaboration, Ukrainian law also prosecutes health care providers who voluntarily take clinical positions in the occupation regime. This offense is a misdemeanor, less severe than assuming a senior administrative position, and does not impose a prison sentence. Nevertheless, the law has ensnared a large number of teachers and some providers of medical or social services. The following two cases serve as examples.

- **Collaboration by service providers 1.** At the Forensic and Medical Bureau in Kherson, an unnamed toxicologist “conducted forensic toxicological examinations, research, and other types of expert work” and advised occupation law enforcement agencies.^55^
- **Collaboration by service providers 2.** A social worker working for the Ingulets village council voluntarily “performed her public service duties by providing information to local residents about social benefits, humanitarian aid, and paid pensions,” in violation of part 2 of article 111‐1 of the Criminal Code of Ukraine.^56^

Although neither case describes patient care, they raise fears that Ukrainian authorities may prosecute caregivers despite official assurances to the contrary. Assuming control of health care, Russian authorities have reorganized and created new facilities, leaving Ukrainian legal experts to worry that a doctor or nurse taking a position in these recently established institutions could face charges of collaboration.^57^ But clinicians are not administrators. Caught up in the vortex of war and occupation and finding themselves at the bedside of a Ukrainian or Russian patient, clinicians should not be expected to refuse treatment to that patient. This fundamental right to health care and its corollary duty to provide beneficent care should be respected in the future, as they have in the past.

However, health care rights have come under fire when some medical workers deny the enemy health care rights. Consider a clear‐cut case of *non*collaboration: Serhii Dragun, the director of a hospital in Ukraine, acknowledged that his staff was disgusted with having to treat captured Russian soldiers before turning them over to military authorities. When asked whether Ukrainian doctors should provide medical aid to wounded Russian service members, he replied that rescuing Russian soldiers might aid in future exchanges or provide valuable information for legal proceedings against crimes and genocide.^58^

Mirroring the Chekhova case (treason 1), Dragun's staff seemed to recognize their prima facie duty to treat their POW patients before delivering them to the military authorities. Nevertheless, one significant difference stands out. A Ukrainian doctor in the occupied territory may consider the possibility that shielded Ukrainian soldiers will recover and then aid their war effort, whether by returning to the Ukrainian lines or remaining in the occupied territory as saboteurs. These military benefits count as a reason to shield such patients, one dictated by civic duty. For a Ukrainian doctor in unoccupied Ukraine, however, the risk that shielded Russian soldiers may imperil Ukrainian interests is a reason to hand them over. Each doctor, therefore, may reach contrasting conclusions as they consider whether to denounce their patients.

This bit of self‐questioning goes unaddressed in the noncollaboration case above. Dragun, as the hospital director, was silent about the medical staff's duty of beneficence and the soldier's right to health care, demonstrating the kind of indifference that often leads to prisoner abuse.^59^ Instead, he framed the staff's motivation to treat Russian soldiers solely in instrumental terms: care for the wounded so that they may furnish intelligence or serve as bargaining chips for future exchanges. This case, in which prisoner‐patients are viewed solely as a means to a military end, indefensibly prioritizes fidelity to the state and the military mission over fundamental health care rights respecting patient dignity. Conditioning POW care on non‐health‐related benefits may further jeopardize treatment if those benefits evaporate. While medical personnel working in their unoccupied home state are not liable to charges of collaboration, they are guided by norms similar to those that apply to personnel working under occupation. In each case, their primary responsibility is to their patients’ welfare. In each instance, military authorities instructed doctors to denounce their patients in the name of military necessity, whether to gain information, use them as bargaining chips (as Dragun proposed prisoner‐patients may be used), or prevent their return to duty in Ukraine (as in the Chekhova case). In each case, a defensible decision depends crucially on ascertaining the safety of their patients if they are turned over to the authorities. Failing to meet this condition undermines defensible decision‐making, while failing to weigh its implications in court undercuts the verdict and sentence.

## From Criminal Collaboration to Humanitarian Cooperation

In a June 2022 interview, Victor Liashko, Ukraine's minister of health, was asked, “How can medical workers [in occupied Ukraine] not become collaborators?”

“Do not cooperate with the Russian occupiers,” he replied. He then elaborated, “All cooperation that ensures the vital functioning of the health care institution will not be considered collaborationism. When a person takes the initiative to secure a managing position, cooperates with the occupation authorities for political reasons, or recognizes the legality of the occupation, it will be regarded as a betrayal of Ukraine.”^60^

Antipathy toward collaborators runs deep in Ukraine, threatening citizens living in occupied territory and scorching practitioners and administrators. Echoing the minister of health, only one‐third of the respondents in a 2023 survey would give amnesty to the heads of schools, hospitals, and communal enterprises, while 58 percent, a bare majority, would consider amnesty for teachers, doctors, and social workers who worked for the occupation authorities.^61^ The government and the public set a high bar for civilians to clear. Anyone unfortunate enough to live under occupation will have to prove themselves. Charges of collaboration are not rare. Through December 31, 2024, the courts decided 327 collaboration cases under part 2 (working for the occupation regime) and 450 cases under part 5 (taking an administrative position in the regime) of article 111‐1 of the Criminal Code of Ukraine, a 40 percent increase in the six months since June 2024.^62^ The number of arrests, investigations, and pending court cases is unknown.

Ukrainian law and public opinion do not make it easy to defend oneself. Hospital administrators and clinicians find themselves cornered. To protect themselves from charges of collaboration, they must continue to “ensure the vital functioning of health care institutions,” as Liashko demands. At the same time, public health officials face criminal prosecution because they tacitly acknowledge the regime's legitimacy as they assume management positions to ensure vital medical services. Rejecting the regime imperils civilian medical care and may pose personal dangers. Supporting the regime may enhance Russian military capabilities and endanger Ukraine. To work through these dilemmas, health care providers often require more information and guidance than is available.

How should the professional community confront the phenomenon of medical collaboration? While some legal scholars and jurists have scrutinized the ambiguities of the current law and the dangers it poses for many public officials in occupied Ukraine, the medical establishment has been reticent about addressing the legal and ethical challenges of medical collaboration. Among the paramount duties of medical associations is providing their members with the information and guidance they require to confront the legal and ethical challenges of medical practice, during both war and peace.^63^

In a survey my team conducted in 2024‐2025 to determine how Ukraine's medical community advised its members to cope with the conflicting legal, ethical, and civic norms dominating their practice under occupation, we contacted thirty‐three Ukrainian medical associations and journals to ask, “What guidance do you offer your members or readers about the duties and dangers of cooperating with the Russian occupation regime, whether as caregivers or health care administrators?”^64^ Among the eighteen who answered, none offered guidance to health professionals, including the *Zaporizhzhia Medical Journal*, whose region remains partly occupied. The Ukrainian Medical Association acknowledged that the topic was “very important” but supplied no further information.

The reluctance of the medical associations and journals to tender professional advice is understandable. The medical community cannot advise its members to break the law and collaborate or to abandon the Ukrainian health care system as it strives to maintain service in occupied Ukraine. To navigate these straits, some legal commentators emphasize the unique place of “humanitarian collaboration.” In contrast to military collaboration, which enhances Russia's armed forces, humanitarian collaboration ensures the health, welfare, and livelihood of the occupied civilian population, paving the way for proposals to impose less severe penalties for humanitarian collaboration.^65^ Nevertheless, humanitarian collaboration, as envisioned, remains a form of illegal, criminal cooperation, subject to prosecution and punishment (albeit less severe than that for military collaboration) and does little to alleviate the dilemmas of providing health care under occupation.

Ensuring the delivery of adequate health care under military occupation requires more than legislation that differentiates between military and humanitarian collaboration. It requires a concerted effort to shed the stigma of collaboration completely. To meet these challenges, a policy of legally endorsed *humanitarian cooperation*, rather than criminal “medical collaboration,” offers a more balanced legislative approach. Consistent with the humanitarian and professional duties of public officials, humanitarian cooperation acknowledges the right of these officials to consult their conscience as they struggle to maintain welfare services during a harsh military occupation.

Conscientious choice judiciously balances the costs and benefits of cooperation and remains the key to ethical decision‐making under occupation. Costs comprise personal endangerment and hazards to the state. Benefits stem from an efficient system that meets the public's health care needs. Humanitarian cooperation recognizes that occupation authorities can support or undermine the ability of health care professionals to maintain adequate health care for the civilian population. When the occupation regime supports efforts to ensure adequate care, local health officials may conscientiously cooperate even at the cost of diverting excess resources (that is, those remaining after ensuring adequate care) to the occupation authorities for military purposes. However, when an abusive regime violates patients’ and workers’ human rights or forces health officials to prioritize resources for Russian soldiers at the expense of essential care for local civilians, these officials have little choice but to conscientiously refuse their cooperation. Acting otherwise invites charges of criminal medical collaboration.

Unfortunately, the current legal status of medical personnel is ambiguous, risky, and counterproductive. Health care professionals should enjoy protection from coercion and prosecution by the occupying forces and their home government, but they do not. Both parties to the conflict routinely disregard these protections. By ignoring the health care officials’ public duties and their right to professional independence, Ukrainian legislation and judicial proceedings overlook the possibility of defensible humanitarian cooperation entirely. Failing to decriminalize humanitarian cooperation puts clinicians and administrators at risk for prosecution, dissuades many working under occupation, and undermines support for postwar reintegration if and when these territories are liberated. At the same time, large‐scale conscientious refusal by medical administrators to cooperate with the Russian occupation jeopardizes public health. It is defensible only if the occupation regime is flagrantly criminal or if health care resource allocation egregiously ignores local needs. Avoiding these consequences is possible only if health care professionals receive the information necessary to competently assess the costs and benefits of cooperation and a clear list of outlawed security activities that could bedevil the provision of health care.

Until the local and international medical communities take a stronger stand to sever “collaboration” from humanitarianism and medicine, health care professionals working in occupied territories remain vulnerable, their legal status fragile and opaque. Historically, Ukraine is exceptionally wary of collaboration and quick to condemn public officials for what amounts to fulfilling their duties to safeguard the public's welfare. As human rights organizations press to distinguish criminal collaboration from conscientious professionalism,^66^ the medical community can follow suit by supporting the concept of humanitarian cooperation and endorsing firm ethical guidelines that enable medical practitioners and health care administrators in occupied territories to fulfill their professional responsibilities without fear of recrimination and prosecution.

## Acknowledgments and a Note about the Ukrainian Sources

I am grateful to Onysiia Syniuk, legal analyst at the Human Rights Center in Kyiv, Ukraine, for providing data, interpretation, and additional sources related to Ukrainian collaboration laws and prosecutions.

The endnotes contain English and Ukrainian sources. The Ukrainian sources were machine‐translated for analysis and are searchable only in Ukrainian. I am indebted to Oleksandra Cherniakhovska for providing comprehensive bibliographical services in English and Ukrainian; communicating with Ukrainian legal, medical, and human rights organizations; verifying the English translation of the in‐text citations; and supplying short descriptions of Ukrainian websites, nongovernmental organizations, and blogs that published some of the referenced articles. These descriptions appear at the end of the relevant citations.

Funding for this project was provided by the Israel Science Foundation, through grant 852/23.
